# Combined clustered scan-based metal artifact reduction algorithm (CCS-MAR) for ultrasound-guided cardiac radioablation

**DOI:** 10.1007/s13246-022-01192-6

**Published:** 2022-11-09

**Authors:** Sathyathas Puvanasunthararajah, Saskia M. Camps, Marie-Luise Wille, Davide Fontanarosa

**Affiliations:** 1 School of Clinical Sciences, Queensland University of Technology, Brisbane, QLD Australia; 2 Centre for Biomedical Technologies, Queensland University of Technology, Brisbane, QLD Australia; 3 EBAMed SA, Geneva, Switzerland; 4 School of Mechanical, Medical & Process Engineering, Faculty of Engineering, Queensland University of Technology, Brisbane, QLD Australia; 5 ARC ITTC for Multiscale 3D Imaging, Modelling, and Manufacturing, Queensland University of Technology, Brisbane, QLD Australia

**Keywords:** Cardiac arrhythmias, Metal artifact reduction, Computed tomography, Ultrasound guidance, And cardiac radioablation

## Abstract

Cardiac radioablation is a promising treatment for cardiac arrhythmias, but accurate dose delivery can be affected by heart motion. For this reason, real-time cardiac motion monitoring during radioablation is of paramount importance. Real-time ultrasound (US) guidance can be a solution. The US-guided cardiac radioablation workflow can be simplified by the simultaneous US and planning computed tomography (CT) acquisition, which can result in US transducer-induced metal artifacts on the planning CT scans. To reduce the impact of these artifacts, a new metal artifact reduction (MAR) algorithm (named: Combined Clustered Scan-based MAR [CCS-MAR]) has been developed and compared with iMAR (Siemens), O-MAR (Philips) and MDT (ReVision Radiology) algorithms. CCS-MAR is a fully automated sinogram inpainting-based MAR algorithm, which uses a two-stage correction process based on a normalized MAR method. The second stage aims to correct errors remaining from the first stage to create an artifact-free combined clustered scan for the process of metal artifact reduction. To evaluate the robustness of CCS-MAR, conventional CT scans and/or dual-energy CT scans from three anthropomorphic phantoms and transducers with different sizes were used. The performance of CCS-MAR for metal artifact reduction was compared with other algorithms through visual comparison, image quality metrics analysis, and HU value restoration evaluation. The results of this study show that CCS-MAR effectively reduced the US transducer-induced metal artifacts and that it improved HU value accuracy more or comparably to other MAR algorithms. These promising results justify future research into US transducer-induced metal artifact reduction for the US-guided cardiac radioablation purposes.

## Introduction

Cardiac arrhythmias are a major global health concern and it has been predicted that atrial fibrillation, which is the most common type of cardiac arrhythmia, will affect 6–12 million people in the USA by 2050 and 17.9 million people in Europe by 2060 [1]. Among currently available curative treatment methods for cardiac arrhythmias, catheter ablation is the most widely used [2]. However, catheter ablation is an invasive procedure which can have a long treatment time, and its success rate can be low due to the inaccessibility of some arrhythmogenic tissues [3].

Cardiac radioablation is a promising treatment method that could potentially overcome the limitations faced by catheter ablation. The primary aim of cardiac radioablation is to treat arrhythmias by noninvasively delivering a radiation dose to the arrhythmogenic tissues using external beam radiation therapy. Among the external-beam radiation therapy, photon and proton beams are investigated for cardiac radioablation in pre-clinical [4–9] and clinical [10–16] research studies.

A cardiac radioablation workflow typically consists of a simulation stage, where the treatment is planned, and of a treatment delivery stage. At the simulation stage, planning computed tomography (CT) scans are acquired and subsequently used to delineate the treatment target (arrhythmogenic tissue), and organs-at-risk (OARs). Then, the electron density information derived from the CT Hounsfield units (HU) is used for the calculation of the radiation dose distributions inside the delineated structures [17]. At the treatment delivery stage, the external beam delivers the treatment dose to the arrhythmogenic tissue, while sparing the OARs as much as possible. However, intra-fractional motion which occurs during the treatment delivery, due to heartbeat and respiration may negatively impact the accuracy of the dose delivery [18, 19]. This makes real-time monitoring of both cardiac and respiratory motion during the treatment of paramount importance.

Ultrasound (US) imaging is a non-ionizing radiation based real-time imaging modality which is clinically used for treatment guidance in radiation therapy of oncological targets [20–22]. US-based treatment guidance is currently being investigated for cardiac radioablation, where it has potential for dose delivery accuracy improvement [23–25]. This type of image guidance relies on US imaging of the cardiac tissue position during the radiation dose delivery in order to compare this position with the corresponding position at the simulation stage based on which the treatment was planned. To reduce the number of workflow steps and the time needed during the simulation stage, the US scan could be acquired simultaneously with the planning CT scans. The presence of the US transducer when the CT scan is acquired, though, can result in transducer-induced metal artifacts on the planning CT scans, which are caused by metal components located inside the US transducer [26, 27].

To reduce the negative impacts of this type of artifacts induced by metal, Metal Artifact Reduction (MAR) algorithms have been investigated and/or proposed in the literature, mainly focusing on the artifacts generated by implanted metal structures. Some of these algorithms are commercially available, and some are only research-based [28]. Many of these algorithms use the principle of sinogram inpainting technique [29]. Sinograms, or in other words projection data, are acquired during the CT scan acquisition and consist of the attenuation profiles of X-ray respective to an angle of the X-ray beam. During the sinogram inpainting technique, the projection data which are affected by metal components are treated as missing values. This technique uses interpolation methods, typically linear interpolation, to “paint” the missing values from surrogate data. When surrogate data are near to metal components or consist of high contrast structures, especially bone, the direct interpolation of a sinogram tends to induce new artifacts [30]. To minimize or avoid the creation of new artifacts, a normalization step is introduced during the sinogram interpolation in the normalized MAR (NMAR) method [31]. NMAR requires information on the regions corrupted by metal artifacts for the artifact reduction. For this, tissue types in the CT scan which are corrupted by metal artifacts (CT_art_) are classified into different clusters to create a clustered scan. The clustered scan has similar content as the CT_art_ scan, but it has less or even no metal artifacts. After generation of the clustered scan, it is forward projected to the sinogram space and used to normalize the sinogram of the CT_art_ scan for the interpolation of missing values. The performance of the NMAR method depends on the quality of clustered scan, which is often affected by the number of metal artifacts.

In general, the application of a MAR algorithm on a CT_art_ scan results in an artifact-corrected CT (CT_cor_) scan. The commercially available MAR algorithms of Orthopaedics Metal Artifact Reduction (O-MAR, Philips Health System, Cleveland, USA) and iterative Metal Artifact Reduction (iMAR, Siemens Healthcare, Forchheim, Germany) were evaluated for photon and proton therapy applications [32–40]. These phantom and clinical studies revealed that the O-MAR and iMAR algorithms could be beneficial for the improvement of therapy treatment planning either in terms of image quality or dosimetric outcome. Research-based MAR algorithms have also been evaluated for radiotherapy applications. In particular the metal deletion technique (MDT) [41], MAR with hardware adaptation [42], and ker MAR [43] have been investigated. It was shown that these algorithms can be used to reduce the errors on photon and proton range calculation during treatment planning [42, 44, 45].

Another possible approach for metal artifact reduction is through the acquisition of a dual-energy CT (DECT) scan instead of a single energy scan. In this method, two discrete energy beams (typically 90 kVp and 140 kVp) are used rather than a polychromatic X-ray beam (average energy of 120 kVp) of a conventional single energy CT (SECT) [46, 47]. The utilization of a discrete high energy beam reduces the beam-hardening effect [48], which is one of the factors that contributes to the creation of metal artifacts on CT scans [49]. In literature, the application of a commercial MAR algorithm, especially iMAR, on both SECT scans and DECT scans was evaluated for metal artifact reduction. Generally, the application of a MAR algorithm on DECT scans better reduced the artifacts than the application of these algorithms on SECT scans [50, 51].

To the best of our knowledge, none of the studies in literature proposed or investigated MAR algorithms for the reduction of US transducer-induced metal artifacts on planning CT scans. Therefore, the aim of this work was to develop a specialized automatic MAR algorithm which can be used to correct these US transducer-induced metal artifacts on SECT and DECT scans. As O-MAR, iMAR and MDT have been widely investigated for photon and proton therapy applications in the literature, the performance of the proposed MAR algorithm has been compared with the performance of these commercial and research-based MAR algorithms.

## Materials and methods

### CCS-MAR algorithm design

CCS-MAR is a sinogram inpainting-based MAR algorithm which combines image processing strategies with the NMAR method [31] to replace artifact corrupted areas in CT scans. The NMAR method requires a CT scan which is clustered according to tissue types as input. The clustering approach utilized in this work was inspired by the studies conducted by Wu et al. [52] and Luzhbin and Wu [53]. These studies utilized the k-means clustering algorithm [54], which is an unsupervised iterative method being used for classification tasks, for the creation of clustered CT scans. As the performance of the NMAR method depends on the quality of the clustered CT scan, the creation of the clustered scan without metal artifacts is crucial. Therefore, the key step of the CCS-MAR algorithm is the creation of an artifact-free clustered CT scan from the CT_art_ scan.

**Fig. 1:**
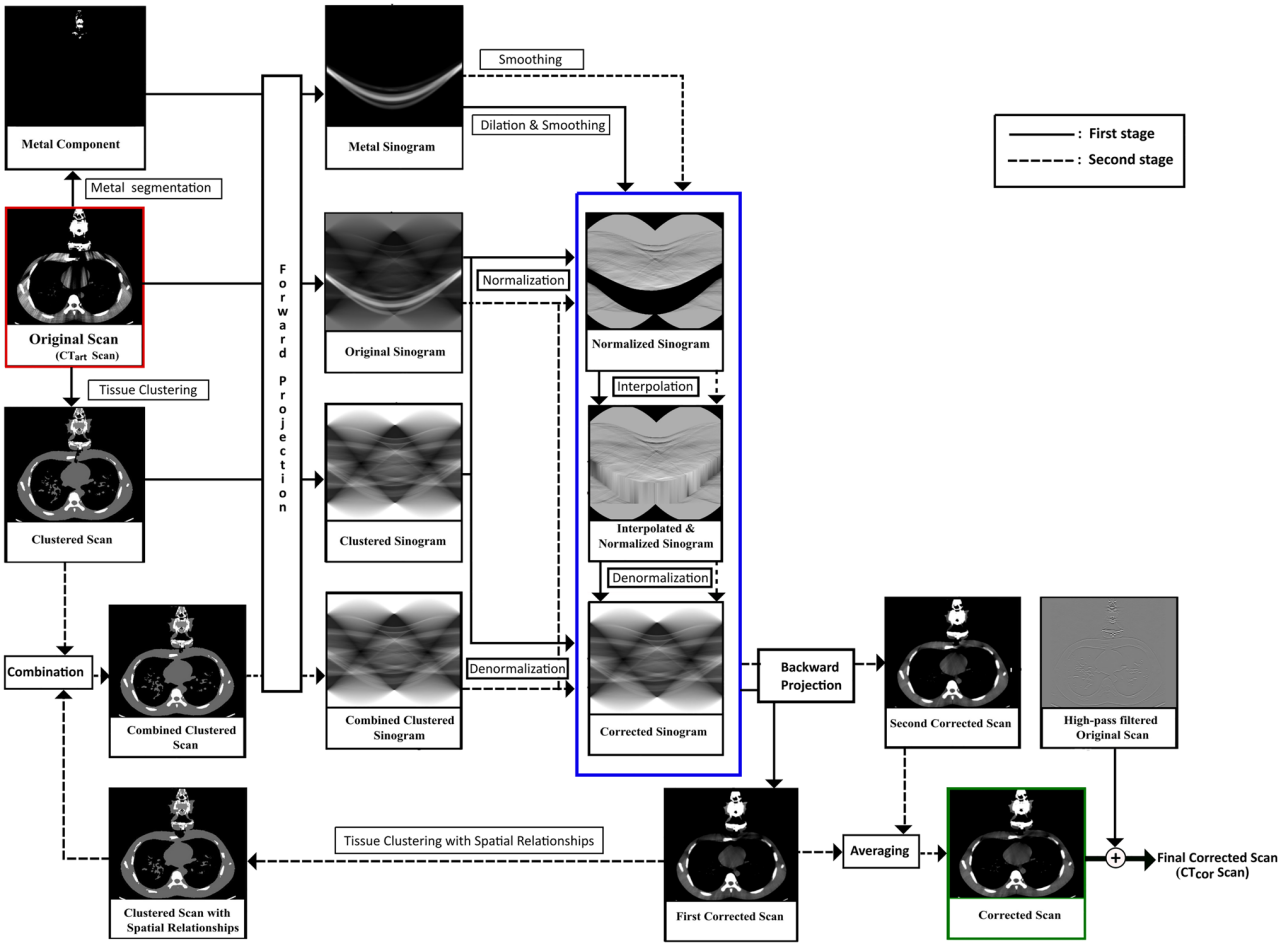
Scheme of the CCS-MAR algorithm and its two-stages. The first and the second correction stages are indicated using black lines and dotted black lines, respectively. Starting from the metal segmentation and tissue clustering, CCS-MAR produces the original, the metal, and the clustered sinograms. Then, NMAR method which is in the blue box is applied to create the first corrected scan. The clustered scan with spatial relationships is created from the first corrected scan and then a combined clustered scan is generated. The NMAR method is applied again to the combined clustered sinogram to create the second corrected scan. From the first and second corrected scans, the corrected scan is created, and then noise texture is added to generate the final corrected scan

The CCS-MAR algorithm was implemented using MatLab (version 9.7, The MathWorks Inc, Natick, MA, USA), and its workflow is shown in Fig. 1. It is a fully automatic algorithm which does not require any manual contouring of artifact-corrupted areas. CCS-MAR utilizes a two-stage correction process for metal artifact reduction. The first stage of correction process and the second stage correction process is indicated using black, and dotted black arrows in Fig. 1, respectively. The input of the algorithm is the planning CT_art_ scan, which will be referred to as ‘original’ scan in the rest of this section. During the first correction process of the algorithm’s workflow, HU value thresholds of 2000 HU and -950 HU are used to identify and segment the metal component (see Fig. 1) and air region, respectively. A fixed threshold value of 2000 HU has been chosen for the metal segmentation based on previous studies published in the literature [55, 56]. However, a slight change in the HU value threshold from 2000 to 2500 HU for the metal segmentation also has been checked. In order to create an initial clustered scan, the k-means clustering algorithm is applied to the original scan after the metal and air segmentation. As the original scan is acquired of the thoracic region, this scan primarily consists of soft tissue, bone, and lung tissue. By considering these three tissue types, a total of three clusters are chosen during the k-means clustering. After the clustering, the segmented air region from the first step is added to the clustered scan. Subsequently, the original scan, the metal component, and the clustered scan are forward projected to generate their respective sinograms.

The metal sinogram is then modified through the application of both dilation with a disk structuring element [57] and smoothing through Gaussian filtering. Since the exact HU value of the metal component in the transducer is unknown, dilation is applied to inflate the metal data to minimize the dependency on the 2000 HU value threshold. The smoothing is applied to remove statistical photon noise which may cause streak artifacts during the reconstruction of CT scans.

In the next step of the algorithm, the NMAR method [31] is applied to the created sinograms (see the blue box in Fig. 1). The main steps of NMAR are normalization, interpolation, and denormalization of the sinograms. In the normalization step, the original sinogram is divided by the clustered sinogram and a normalized sinogram is created. Then, the dilated and smoothed metal sinogram is used as a mask to identify the metal region in the normalized sinogram for the linear interpolation. Subsequently, the interpolated normalized sinogram is multiplied by the clustered sinogram during the denormalization step resulting in a corrected sinogram. Filtered back projection is finally used to reconstruct the first corrected CT scan from the corrected sinogram.

Even though most of the artifacts are reduced in the first corrected scan, typically the intense dark streaks which are in the region close to the transducer remain unchanged. Also, the HU value of this region becomes lower than the HU value of the corresponding region on the original scan, because the intense dark streaks are modelled as lung tissue in the clustered scan. Therefore, further correction is required, for this, the second stage which is the final correction process is incorporated to reduce the errors in the first corrected scan. In the beginning of the second correction process, the pixel-wise absolute difference between the original scan and the first corrected scan is calculated. An empirically chosen threshold value of 200 HU is used to identify the pixels which are in the region with the dark streaks. Subsequently, those pixels are identified on the first corrected scan, and they are replaced with a 0 HU value. A value of the threshold in the range of 150 HU to 200 HU is suitable for the HU value replacement. Indeed, it was found that the threshold value below 150 HU will replace the HU values of pixels which do not have metal artifacts on the first corrected scan. This contributes to the generation of an inaccurate second clustered scan. On the other hand, a value above 200 HU will not contribute to the artifact-free combined clustered scan. The region with intense dark streaks may cause a wrong classification during k-means clustering. Thus, spatial relationships [52] are incorporated with k-means clustering, calculated as:

1 $$C_{i} = \arg \left\{ {\max \left( {\left| {P \cap C_{j} } \right|} \right)} \right\}, j = 1, \ldots k$$

where $P$ denotes a set of pixels covered by a 3 × 3 mask centred around pixel $i$, and $|.|$ is the number of members in the set after the k-means clustering. ${C}_{j}$ and $k$ are the $j$^th^ cluster and the total number of clusters, respectively. During this procedure, pixel $i$ is reassigned to the cluster ${C}_{i}$ that has the maximum number of members within the mask. With this method, the clustered scan with spatial relationships is created, and then the air region which was segmented in the first step is added. For the mask size, the minimum size of 3 × 3 produced the best result for incorporating spatial relationships. On the other hand, the mask with a larger size smeared the clustered scan, which would then produce the second corrected scan with induced secondary artifacts. Even though incorporating spatial relationships allows to reduce the inaccuracies in clustering, especially in the artifact regions, it may also induce errors in pixel reassignment in the artifact-free regions. For this reason, during the initial k-means clustering, the spatial relationships are not incorporated.

In the next step, the clustered scans which are created with and without incorporating the spatial relationship are combined to generate the artifact-free combined clustered scan. For the combination, the absolute difference between those scans is calculated and the difference is added to the initial clustered scan to reduce the intense dark streaks in the region close to the US transducer. Afterwards, the combined clustered scan is forward projected to generate the combined clustered sinogram. Utilizing the combined clustered sinogram, the original sinogram, and the smoothed metal sinogram, the second corrected scan is reconstructed using filtered back projection after the NMAR method. The dilation of the metal sinogram may cause over interpolation or blurring especially in the region which is adjacent to the US transducer in the second corrected scan. In order to balance this effect, during the creation of second corrected scan, only smoothing and no dilation is applied to the metal sinogram. The corrected scan is an average of the first and second corrected scans. To preserve the noise texture, the result from the application of a high-pass filter on the original scan is added to the corrected scan for the creation of the final corrected scan (CT_cor_ scan).

### CT scan data collection for performance evaluation

**Fig. 2:**
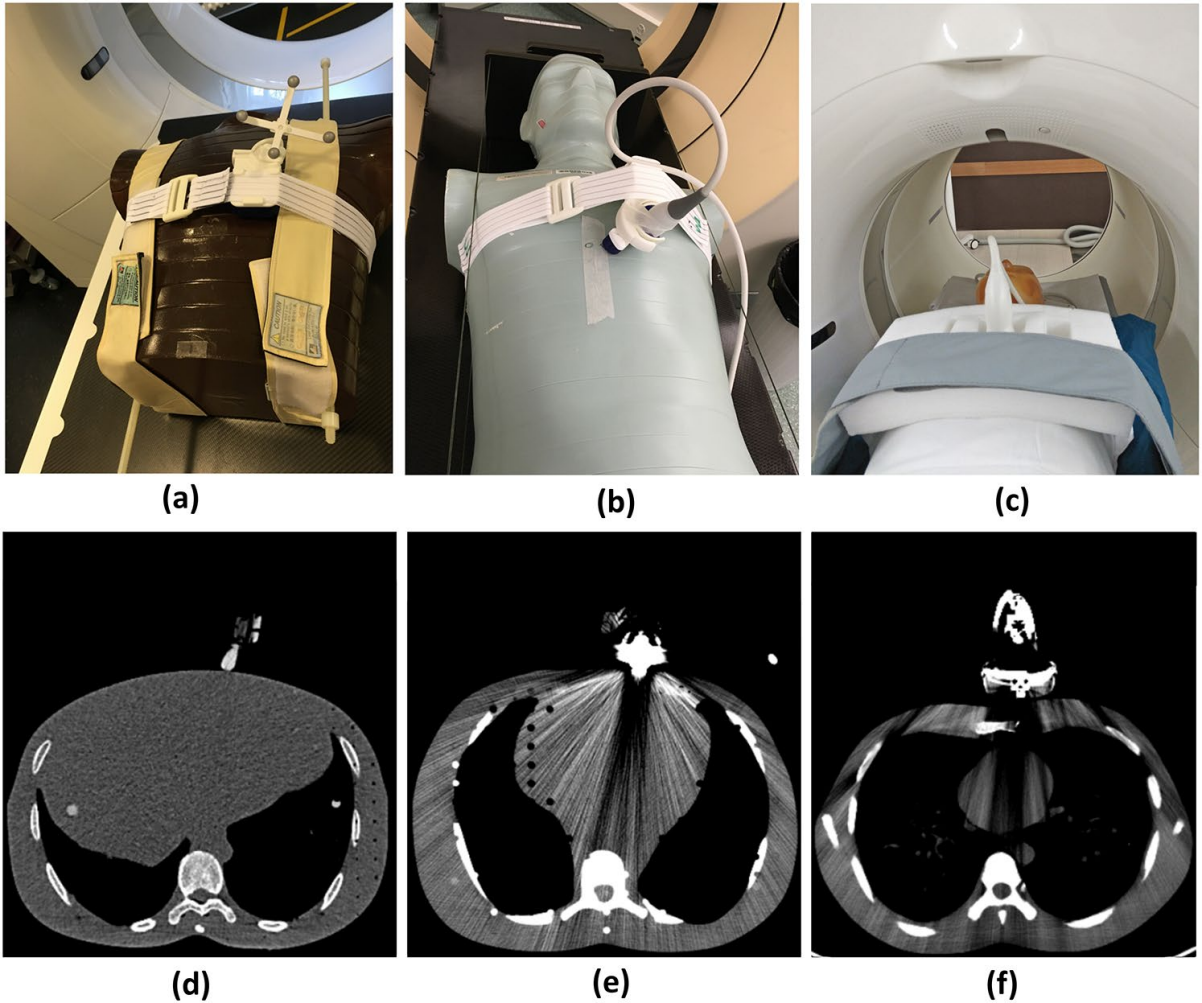
Positioning of the anthropomorphic phantoms for CT scanning and their respective CT scans. **a** ART phantom with the transducer holder in place. **b** ATOM® phantom, and **c** CT torso phantom with a US transducer positioned into the transducer holder. Respective CT scans resulting from CT scanning the setups as shown in **a**, **b** and c are given in **d**, **e**, and **f**

CT scanning and acquisition parameters are summarized in Table 1. In total, three types of adult anthropomorphic phantoms were used: a Model ART-211 male phantom (ART, Radiology Support Devices, Long Beach, CA, USA), an ATOM® male phantom (CIRS, Model-701, Norfolk, VA, USA), and a CT torso phantom (CT Torso, Model CTU-41, Kyoto Kagaku Ltd, Japan). The phantoms were positioned on the CT table in a head-first position (Fig. 2) and scanned first without, and then with US transducers in place. As can be seen in Table 1, three different CT scanners from Philips and Siemens were used for CT scanning.

**Table 1:** Details of utilized CT scanners, clinical site, scan types (SECT, single energy CT; DECT, dual-energy CT), anthropomorphic phantoms, US transducers and CT scanning parameters

CT scanner (model)	Siemens CT (SOMATOM definition AS)	Philips CT (brilliance big bore)	Siemens PET-CT (biograph 128)
Clinical site	Kantonnspital Aarau, Aarau, Switzerland	Geneva University Hospital, Geneva, Switzerland	Herston Imaging Research Facility (HIRF), Brisbane, Australia
Scan type	SECT and DECT	SECT	SECT
Phantom	ART	ATOM®	CT torso
US transducer	Single-plane phased array, Bi-plane phased array	Single-plane phased array, Bi-plane phased array	Linear volume array
Tube current	120 kVp (SECT) 80 kVp/140 kVp (DECT)	120 kVp	120 kVp
Field of view (FOV)	500 mm	600 mm	500 mm
Slice thickness	1 mm	1 mm	2 mm
Scan dimension	512 × 512	512 × 512	512 × 512
Pitch factor	1	1	1
Reconstruction kernel	Body (Br38f)	Sharp (C)	Body (B31f)

The size of an US transducer influences the amount and the appearance of metal artifacts on CT scans [26]. To investigate this effect, a total of three US transducers with various sizes were used: (1) single-plane phased array: Telemed P5-1S15-A6 from Telemed (UAB, Vilnius, Lithuania); (2) bi-plane phased array: Terason XY mini from Teratech Corporation (Burlington, MA, USA); and (3) linear volumetric array: Philips VL13-5 from Philips Healthcare (Andover, MA, USA). The maximum width of the metal part inside the transducers as measured on CT scans of above transducers were 4 cm, 2 cm, and 6 cm, respectively. To fix the US transducer on the phantoms, custom-built transducer holders (consisting of 3D printed plastic parts and elastic straps) which were developed in collaboration with Usono (Eindhoven, the Netherlands) were used.

For each phantom, first a CT scan was acquired without the transducer on the phantom, which resulted in a reference artifact-free CT (CT_ref_) scan. Then, without changing the position of the phantom, an US transducer was placed into the transducer holder and a CT scan with the US transducer-induced metal artifacts (CT_art_) was acquired. The transducers were placed in a position considered to be suitable for imaging the heart. For all CT scans, a CT thorax protocol was utilized, and the scanning parameters are given in Table 1.

The commercial MAR algorithms iMAR and O-MAR were applied during the CT scan reconstruction on the scanner, as these algorithms are available on Siemens and Philips scanners, respectively. iMAR was not applied to the Siemens PET-CT scans, because it was not available on this particular scanner. In addition to the application of these commercial MAR algorithms, MDT and CCS-MAR algorithms were also applied to all CT_art_ scans.

### Image quality metrics analysis

In order to evaluate the effectiveness of a MAR algorithm for metal artifact reduction, image quality metrics including, structural similarity (SSIM) index, root mean square error (RMSE) of the HU values, and peak signal-to-noise ratio (PSNR) [58, 59] were calculated between the CT_ref_ scan and corresponding CT_art_ and CT_cor_ scans. To not bias these image quality metric results, pixels containing the details of the US transducer on CT_art_ scans were copied and transferred to the corresponding CT_ref_ scans. Higher SSIM which ranges from 0 to 1, and higher values of PSNR indicate better image quality. On the other hand, the calculated RMSE values should be low for better artifact reduction. In the end, mean values of these image quality metrics calculated for all the CT_art_ and CT_cor_ scans were used for analysis.

### HU value restoration evaluation

**Fig. 3:**
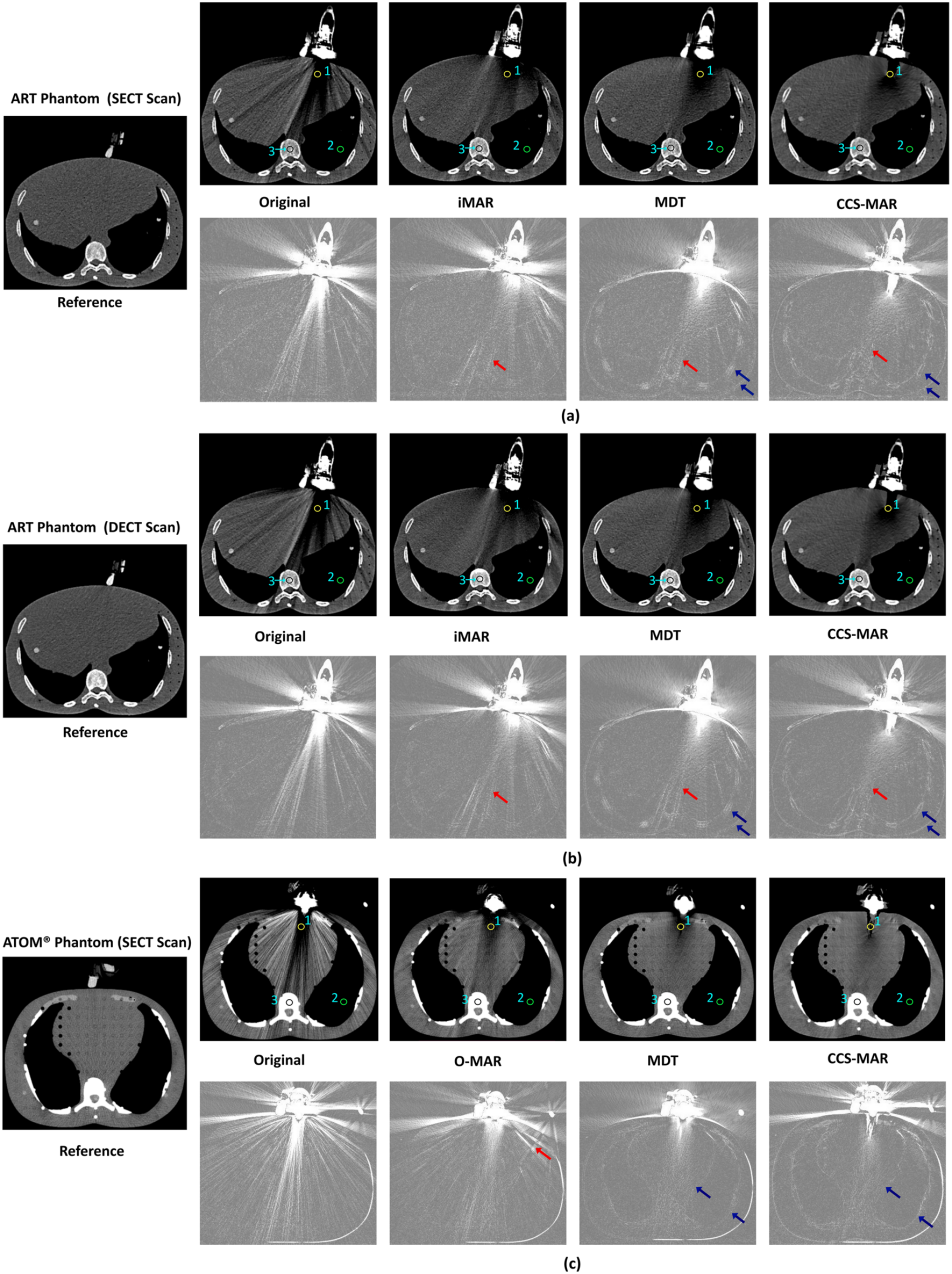
SECT (**a**) and DECT (**b**) scans of the ART phantom, and SECT scans of the ATOM® phantom (**c**) with and without the single-plane phased array transducer placed into the transducer holder. In **a**, **b** and **c**, the upper images from left to right show: the CT scan with the US transducer in place (original CT_art_ scan), and the artifact corrected (CT_cor_) scans after iMAR (**a** and **b**), O-MAR (**c**), MDT, and CCS-MAR application, respectively [Window level/ width: 50/ 350]. The scan on the very left-hand side is the reference (CT_ref_) scan without a US transducer, but with a transducer holder in place. The bottom images show: the absolute difference between the upper CT scans and the CT_ref_ scan [window level/ width: -200/ 200]. The positions of the ROI (1: heart, 2: lung, 3: bone) for the HU value measurements are shown in CT_art_ scan, and in the respective CT_cor_ scans. The red arrows indicate induced secondary artifacts, while the blue arrows indicate edge modifications

**Fig. 4:**
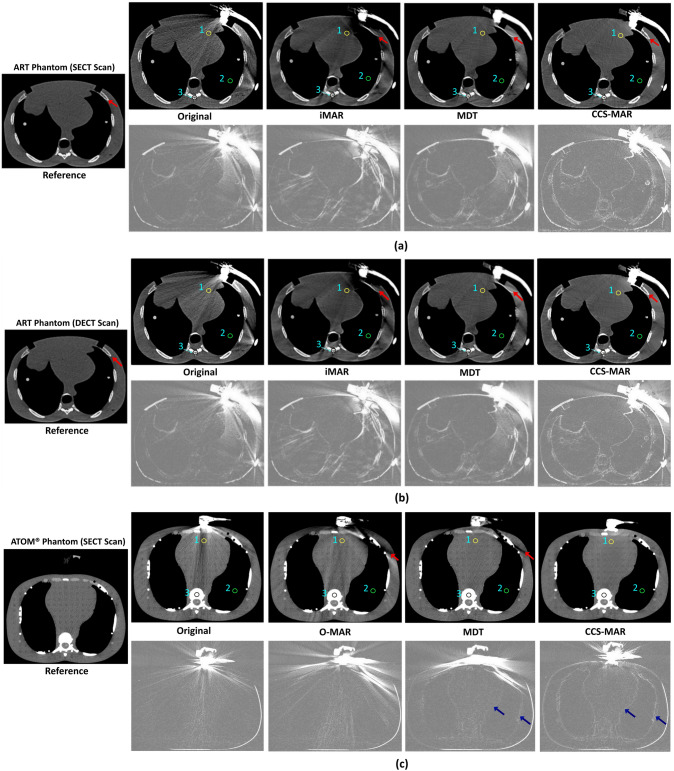
SECT (**a**) and DECT (**b**) scans of the ART phantom, and SECT scans of the ATOM® phantom (**c**) with and without the bi-plane phased array transducer placed into the transducer holder. In **a**, **b** and **c**, the upper images from left to right show: the CT scan with the US transducer in place (original CT_art_ scan), and the artifact corrected (CT_cor_) scans after iMAR (**a** and **b**), O-MAR (**c**), MDT, and CCS-MAR application, respectively [Window level/ width: 50/ 350]. The scan on the very left-hand side is the reference (CT_ref_) scan without a US transducer, but with a transducer holder in place. The bottom images show: the absolute difference between the upper CT scans and the CT_ref_ scan [window level/ width: -200/ 200]. The positions of the ROI (1: heart, 2: lung, 3: bone) for the HU value measurements are shown in CT_art_ scan, and in the respective CT_cor_ scans. The red arrows in **a** and **b** shows bone details, and in c indicate induced secondary artifacts while the blue arrows indicate edge modifications

**Fig. 5:**
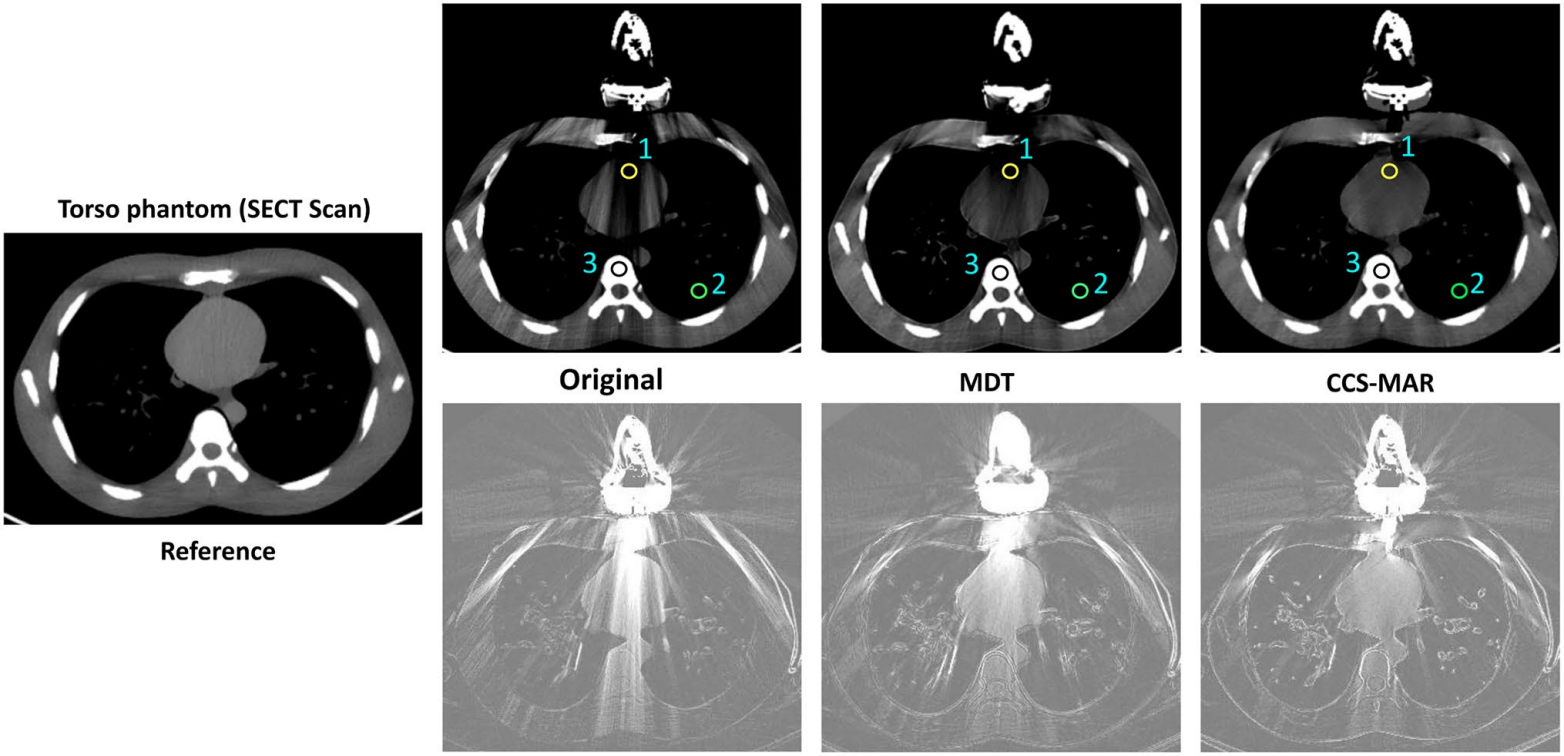
CT scans of the CT torso phantom with and without the linear volume array transducer placed into the transducer holder. The upper images from left to right show: the CT scan with the US transducer in place (original CT_art_ scan), and the artifact corrected (CT_cor_) scans after MDT, and CCS-MAR application, respectively [Window level/ width: 50/ 350]. The scan on the very left-hand side is the reference (CT_ref_) scan without a US transducer. The bottom images show: the absolute difference between the upper CT scans and the CT_ref_ scan [Window level/ width: -200/ 200]. The positions of the ROI (1: heart, 2: lung, 3: bone) for the HU value measurements are shown in the CT_art_ scan, and in the respective CT_cor_ scans

Quantitative comparisons were performed to evaluate HU values on the CT_art_ and CT_cor_ scans in comparison with the CT_ref_ scan. Initially, through visual inspection, axial CT slices, which were affected by metal artifacts were chosen from each CT_art_ scan. Then, the corresponding axial CT slices were selected from the CT_ref_ and CT_cor_ scans. Regions of interest (ROI) were defined as elliptical areas of ≈ 0.5 cm^2^ and the ROI-based HU values of pixels were extracted from the regions of the heart, lung, and bone from those selected CT slices. Figures 3, 4 and 5 shows the ROI placements. A paired sample t-test was used to compare the ROI-based HU value measurements of pixels on both CT_art_ and CT_cor_ with CT_ref_ slice at a significance level of 0.05.

## Results

### Visual comparison of the CT scans

#### Single-plane phased array transducer

Figure 3 shows an example of 2D slices from the SECT (a) and DECT (b) scans of the ART phantom, and SECT scans of the ATOM® phantom (c) with and without the single-plane phased array transducer in place. Overall, the application of MAR algorithms reduced the metal artifacts on the corresponding CT_cor_ scans. However, residual artifacts were observed on CT_cor_ scans after the application of iMAR and O-MAR (see red arrows in Fig. 3). In addition, a modification in the edges of soft tissues and bone can be seen on the corresponding absolute differences of the CT_cor_ scan after the application of MDT and CCS-MAR algorithms (see blue arrows in Fig. 3).

#### Bi-plane phased array transducer

For the bi-plane phased array transducer, the application of MAR algorithms on SECT and DECT scans of the ART phantom, and SECT scans of the ATOM® phantom are shown in Fig. 4. Due to its smaller size, the bi-plane phased array transducer produced fewer metal artifacts than the single-plane phased array transducer (see previous section). In addition, CCS-MAR better preserved the bone details than the iMAR and MDT algorithms (see red arrows in ‘b’ Fig. 4). However, the application of CCS-MAR and MDT and algorithms slightly modified the edges of soft tissues and bones (see blue arrows in ‘c’ Fig. 4).

#### Linear volume array transducer

Figure 5 shows the CT scans of the torso phantom with and without the volume array transducer. A commercial MAR algorithm was not applied to reduce the metal artifacts on this torso phantom scans since it was not available on the particular CT scanner used for CT scanning.

### Image quality metrics

#### Single-plane phased array transducer

For the single-plane phased array transducer, the image quality metrics SSIM, RMSE and PSNR were calculated for the CT scans of the ART and the ATOM® phantom (see Table 2). Overall, the application of MAR algorithms on the CT_art_ scans improved SSIM and PSNR, while reducing the RMSE values. This shows that the metal artifact reduction had the desired effect on the improvement of image quality and HU values,

**Table 2:** Mean values of SSIM, RMSE, and PSNR for the SECT and DECT scans of the ART phantom and SECT scans of the ATOM phantom® with the single-plane phased array transducer in place. Better values are highlighted in bold

Image quality metrics	Phantom (scan)	Original	iMAR	O-MAR	MDT	CCS-MAR
Mean						
SSIM	ART (SECT)	0.65	0.85	*N/A*	0.85	**0.86**
	ART (DECT)	0.67	**0.87**	*N/A*	**0.87**	**0.87**
	ATOM®	0.61	*N/A*	0.83	**0.84**	**0.84**
RMSE	ART (SECT)	87.94	33.48	*N/A*	32.97	**31.21**
	ART (DECT)	85.27	**32.06**	*N/A*	33.34	32.22
	ATOM®	112.90	*N/A*	59.08	**57.62**	58.73
PSNR (dB)	ART (SECT)	16.41	24.99	*N/A*	25.11	**25.60**
	ART (DECT)	18.84	28.27	*N/A*	28.27	**28.41**
	ATOM®	18.56	*N/A*	26.12	**26.74**	26.31

*SECT* single energy computed tomography, *DECT* dual-energy computed tomography, *N/A* not applicable

For the CT scans of ART phantom and ATOM® phantom, the calculated mean SSIM values were well improved after the application of CCS-MAR algorithm. The mean RMSE values for the CT_art_ scans of the SECT and DECT scans of ART phantom, and the ATOM® phantom scans were 87.94, 85.27, and 112.90, respectively, and these values were reduced to the lowest after the application of CCS-MAR (31.21) and iMAR (32.06) and the MDT (57.62) algorithms, respectively. The highest mean PSNR (dB) values were observed for the CT_cor_ scans after the application of CCS-MAR for both SECT and DECT scans of the Atom phantoms.

#### Bi-plane phased array transducer

Table 3. shows the image quality metrics for the ART and the ATOM® phantom scans with the bi-plane phased array transducer. On the ATOM® phantom scans, the CT_cor_ after the application of CCS-MAR (29.96) had lower mean RMSE values than the CT_cor_ scans after the application of O-MAR (42.52) and MDT (32.33) algorithms. For the same phantom, the mean PSNR (dB) value for the CT_art_ scan was 19.02 and it improved to 29.02, 31.03 and 31.38 after the application of O-MAR, MDT, and CCS-MAR algorithms, respectively.

**Table 3:** Mean values of SSIM, RMSE and PSNR for the SECT and DECT scans of the ART phantom and SECT scans of the ATOM phantom® with the bi-plane phased array transducer in place. Better values are highlighted in bold

Image quality metrics	Phantom (scan)	Original	iMAR	O-MAR	MDT	CCS-MAR
Mean						
SSIM	ART (SECT)	0.73	0.90	*N/A*	**0.91**	**0.91**
	ART (DECT)	0.74	**0.85**	*N/A*	0.83	**0.85**
	ATOM®	0.71	*N/A*	0.86	**0.95**	0.94
RMSE	ART (SECT)	63.18	34.93	*N/A*	33.17	**32.65**
	ART (DECT)	61.84	**31.28**	*N/A*	32.01	31.67
	ATOM®	73.80	*N/A*	42.52	32.33	**29.96**
PSNR (dB)	ART (SECT)	19.47	28.27	*N/A*	**28.77**	28.76
	ART (DECT)	21.34	31.88	*N/A*	**32.63**	32.11
	ATOM®	19.02	*N/A*	29.02	31.03	**31.38**

*SECT* single energy computed tomography, *DECT* dual-energy computed tomography, *N/A* not applicable

#### Linear volume array transducer

The calculated image quality metrics for the torso phantom scans with the volume array transducer in place are shown in Table 4. The application of the CCS-MAR on the CT_art_ scans improved the mean SSIM and PSNR values better than the MDT algorithm while reducing the mean RMSE values. The mean RMSE value for the CT_art_ scan was 114.12 and it was reduced to 63.72 and 55.67 after the application of MDT and CCS-MAR algorithms, respectively.

**Table 4:** Mean values of SSIM, RMSE and PSNR for the SECT and DECT scans of the ART phantom and SECT scans of the ATOM phantom® with the linear volume array transducer in place. Better values are highlighted in bold

Image quality metrics	Original	MDT	CCS-MAR
Mean			
SSIM	0.60	0.81	**0.84**
RMSE	114.12	63.72	**55.67**
PSNR (dB)	18.56	26.31	**26.74**

### HU value restoration evaluation

#### Single-plane phased array transducer

The ability of a MAR algorithm to restore the HU value on the ART and ATOM® phantom scans is given in Table 5. Even though the MAR algorithms improved the ROI-based mean HU value measurements on the phantom scans, significant differences (p < 0.05) were still identified, especially in ROI 1 and ROI 3 between the CT_cor_ scan and the CT_ref_ scan.

**Table 5:** The ROI-based mean (± STD) Hounsfield unit value measurements on SECT and DECT scans of the ART phantom and SECT scans of the ATOM phantom® without and with the single-plane phased array transducer in place

ROI	Phantom (scan)	Reference	Original	iMAR	O-MAR	MDT	CCS-MAR
Mean (± STD) HU							
1	ART (SECT)	− 20.96 (± 19)	− 186.36 (± 78)	− 100.49 (± 29)	*N/A*	− 97.67 (± 26)	**− 92.51 (± 27)**
	ART (DECT)	− 20.44 (± 14)	− 142.75 (± 85)	**− 83.51 (± 26)**	*N/A*	− 100.97 (± 34)	− 87.50 (± 33.41)
	ATOM®	25.08 (± 16)	− 179.40 (± 77)	*N/A*	− 63.20 (± 56)	− 54.04 (± 35)	**− 49.37(± 34)**
2	ART (SECT)	− 605.58 (± 20)	− 614.87 (± 33)	− 619.70 (± 36)*	*N/A*	− 610.85 (± 29)*	− 610.07 (± 29)*
	ART (DECT)	− 611.34 (± 18)	− 624.79 (± 25)	− 612.26 (± 25)*	*N/A*	− 612.35 (± 19)*	− 612.05 (± 21)*
	ATOM®	− 796.07 (± 17)	− 789.45 (± 26)	*N/A*	− 796.24 (± 22)*	− 797.17 (± 14)*	− 795.50 (± 18)*
3	ART (SECT)	138.96 (± 56)	111.21 (± 57)	120.18 (± 59)	*N/A*	**123.69 (± 57)**	122.07 (± 54)
	ART (DECT)	124.23 (± 59)	79.88 (± 61)	123.00 (± 63)*	*N/A*	125.07 (± 62)*	123.08 (± 61)*
	ATOM®	786.53 (± 38)	771.06 (± 48)	*N/A*	775.60 (± 43)	775.14 (± 38)	**777.51 (± 41)**

In each case the CT scan resulting in the smallest difference in ROI-based HU value measurement compared with the reference CT scan is highlighted in bold. Insignificant p-values (> 0.05) are indicated by asterisks (*). Note that insignificant p-values indicate better performance

*ROI* region of interest, *SECT* single energy computed tomography, *DECT* dual-energy computed tomography, *STD* standard deviation, *N/A* not applicable

In the case of the ATOM® phantom scans, for the ROI 1, the application of CCS-MAR algorithm better reduced the difference between the HU value measurement on CT_ref_ and CT_cor_ scans. For these scans, the measured HU value (mean ± STD) on CT_art_ scan was -179.40 ± 77 HU and it improved to -63.20 ± 56 HU, -54.04 ± 35 HU, and -49.37 ± 34 HU on the CT_cor_ scan after the application of O-MAR, MDT and CCS-MAR, respectively, while it was 25.08 ± 16 HU on the corresponding CT_ref_ scan. The analysis of HU in the ROI 3 depicted that the application of MDT on both the SECT and DECT scans of the ART phantom, and the application of CCS-MAR on the ATOM® phantom scans performed well in terms of HU value restoration.

#### Bi-plane phased array transducer

Table 6 summarises the comparison of the ROI-based mean HU values of the ART and the ATOM® phantom scans with and without the bi-plane phased array transducer. In this study, ROI 1 was placed in the heart region and therefore it was assumed to be the target. On the other hand, ROI 2 and ROI 3 were placed in lung and bone regions, respectively. For all ROI measurements on the SECT and DECT scans of the ART phantom, the MDT and CCS-MAR algorithms brought the mean HU value closer (p > 0.05) to the mean HU values on the CT_ref_ scan. In ROI 1 on ATOM® phantom scans, significant differences (p < 0.05) were identified on the HU value measurements on CT_cor_ after the application of the O-MAR algorithm (5.04 ± 28 HU, [mean ± STD]), compared to the CT_ref_ scan (25.94 ± 16 HU, [mean ± STD]).

**Table 6:** The ROI-based mean (± STD) Hounsfield unit value measurements on SECT and DECT scans of the ART phantom and SECT scans of the ATOM phantom® without and with the single-plane phased array transducer in place

ROI	Phantom (scan)	Reference	Original	iMAR	O-MAR	MDT	CCS-MAR
Mean (± STD) HU							
1	ART (SECT)	− 20.55 (± 16)	− 8.30 (± 24)	− 22.00 (± 15)*	*N/A*	− 21.80 (± 11)*	− 18.33 (± 11)*
	ART (DECT)	− 20.38 (± 12)	− 3.59 (± 20)	− 21.47 (± 14)*	*N/A*	− 18.93 (± 11)*	− 19.13 (± 12)*
	ATOM®	25.94 (± 16)	− 32.52 (± 43)	*N/A*	5.04 (± 28)	27.36 (± 17)*	24.78 (± 16)*
2	ART (SECT)	− 605.28 (± 20)	− 582.93 (± 43)	− 594.45 (± 21)	*N/A*	− 607.17 (± 23)*	− 607.34 (± 14)*
	ART (DECT)	− 611.43 (± 18)	− 593.21 (± 11)	− 612.57 (± 17)*	*N/A*	− 611.8 (± 21)*	− 610.61 (± 13)*
	ATOM®	− 795.55 (± 13)	− 792.52 (± 16)	*N/A*	− 795.50 (± 15)*	− 793.89 (± 13)*	− 794.81 (± 12)*
3	ART (SECT)	135.74 (± 54)	130.15 (± 58)	134.08 (± 56)*	*N/A*	136.06 (± 55)*	134.62 (± 54)*
	ART (DECT)	122.37 (± 59)	117.02 (± 57)	121.59 (± 58)*	*N/A*	122.55(± 58)*	121.68 (± 57)*
	ATOM®	749.49 (± 36)	744.81 (± 53)	*N/A*	748.34 (± 40)*	750.06 (± 35)*	748.95 (± 33)*

In each case the CT scan resulting in the smallest difference in ROI-based HU value measurement compared with the reference CT scan is highlighted in bold. Insignificant p-values (> 0.05) are indicated by asterisks (*). Note that insignificant p-values indicate better performance

*ROI* region of interest, *SECT* single energy computed tomography, *DECT* dual-energy computed tomography, *STD* standard deviation, *N/A* not applicable

#### Linear volume array transducer

The comparison of ROI-based HU values on the CT scans of the torso phantom with and without the linear volume array transducer is given in the Table 7. Even though MDT and CCS-MAR improved the ROI-based mean HU value on the CT_cor_ scan compared to the CT_art_ scan, significant differences (p < 0.05) in HU value measurements were still observed between the CT_cor_ and CT_ref_ scans, especially in ROI 1 and ROI 3. However, in ROI 2, the mean HU value (mean ± STD) was improved after the application of MDT (-927.43 ± 120 HU) and CCS-MAR (-928.09 ± 116 HU), and significant differences were not identified with the measurement on the CT_ref_ scan (-926.02 ± 119 HU).

**Table 7:** The ROI-based mean (± STD) Hounsfield unit value measurements on the CT scans of the Atom® phantom without and with the linear volume array transducer in place

ROI	Reference	Original	MDT	CCS-MAR
Mean (± STD) HU				
1	42.44 (± 10)	− 265.11 (± 61)	− 120.27 (± 23)	**− 97.80 (± 11)**
2	− 926.02 (± 119)	− 942.63 (± 130)	− 927.43 (± 120)*	− 928.09 (± 116)*
3	378.37 (± 32)	268.42 (± 90)	**326.65 (± 39)**	319.56 (± 40)

In each case the CT scan resulting in the smallest difference in ROI-based HU value measurement compared with the reference CT scan is highlighted in bold. Insignificant p-values (> 0.05) are indicated by asterisks (*). Note that insignificant p-values indicate better performance

*ROI* region of interest, *STD* standard deviation

Overall, the application of the CCS-MAR algorithm on the CT_art_ scans effectively reduced the metal artifacts and improved the SSIM and PSNR values for all phantoms scans. Also, CCS-MAR better improved the HU values compared to the other MAR algorithms in most of the scenarios. HU value threshold for the metal segmentation is crucial for the performance of a MAR algorithm. For CCS-MAR, it has been verified that slight changes (range 2000–2500 HU) in the HU threshold value did not affect its performance for the metal artifact reduction. Since CCS-MAR does not utilize many iterations during the artifact reduction, for the investigated CT scans which included severe metal artifacts, the algorithm written in MatLab took an average of 1.5 min per CT slice on a i7-8665U, 2.11 GHz CPU. Therefore, for an average of 40 CT_art_ slices, CCS-MAR is estimated to take about an hour to perform the artifact reduction task on a standard personal computer.

## Discussion

In this work, a fully automated algorithm (CCS-MAR) was proposed which can potentially be used to reduce the negative impact of metal artifacts appearing on planning CT scans used during US-guided cardiac radioablation. Furthermore, the performance of the CCS-MAR algorithm for metal artifact reduction and HU value restoration has been compared to other commonly used commercial and research-based MAR algorithms.

In order to check the robustness of the CCS-MAR algorithm, this study utilized CT scans of different anthropomorphic phantoms. These phantoms were scanned with multiple types of CT scanners while US transducers with various sizes were or were not in place during the acquisition. The presented results show that, the CCS-MAR algorithm effectively improved the image quality metrics SSIM and PSNR and that it improved the ROI-based HU values accuracy more or comparably to other MAR algorithms. Also, the application of CCS-MAR induced fewer secondary metal artifacts on CT_cor_ scan than the application of O-MAR, iMAR and MDT (see Figs. 3, 4). However, slight modification of the edges of the bone and soft tissues were identified on CT_cor_ scan after the application of the CCS-MAR algorithm (see Figs. 3, 4, and 5). CCS-MAR utilizes a CT_art_ scan to generate the original sinogram for the metal artifact reduction, while commercial MAR algorithms typically use the original sinogram directly from the CT scanner. This is a possible reason for these edge modifications.

The mean absolute differences of torso phantom scans show notable differences (Fig. 5), especially in the edges of the bone and soft tissues. These differences might be due to the large number of metal artifacts produced from the linear volume array transducer, or phantom movement between the acquisition of CT_ref_ and CT_art_ scans. A lot of effort was put into not moving the phantom during CT scan acquisition; however, the occurrence of a small motion cannot be excluded. It has to be noted that the same phenomena were not observed or to a lesser extent for the other phantoms scans (Figs. 3, 4). Therefore, we hypothesize that it is more likely that the differences originate from the large number of artifacts. In order to draw final conclusion, additional evaluation is required.

It has been shown in literature that the size of a US transducer influences the creation of the metal artifacts [26]. In our case, the CT scans with the single-plane phased array transducer had higher calculated RMSE than the bi-plane phased array transducer. This strengthens the recommendation to use the smallest US transducer possible and subsequently apply an MAR algorithm to reduce the artifacts.

Generally, the application of the MAR algorithms on the DECT scans of the ART phantom with the bi-plane phased array transducer better reduced the RMSE value and better restored the HU value than the application of MAR algorithms on SECT scans of the ART phantom. These results align with the work conducted by Kovacs et al*.* [51] in which they reported that the application of MAR algorithms on DECT scans is the preferred choice to reduce the metal artifacts, especially for the metal artifacts created by dental, and hip implants, for radiotherapy applications. Thus, to further reduce the metal artifacts which are created by the smallest US transducer, the acquisition of DECT scan is recommended over the acquisition of a SECT scan.

This work has a few limitations which are related to the use of CT phantoms for the MAR algorithm evaluation. These phantoms are just simulations of human anatomies and therefore do not contain structures which are specific for cardiology patients, such as pacemakers and cardioverter defibrillators (ICDs). These implantable cardiac electronic devices often consist of metal parts, and therefore they also can produce metal artifacts on CT scans. This study focused solely on the artifacts produced by the US transducer and did not take into account the metal artifacts produced by the implantable cardiac devices itself. In addition, the phantoms used in this work could not be imaged with US. For this reason, the positioning location and angle of the US transducers on the phantoms was chosen based on educated guess. In order to draw final conclusions regarding the performance of the proposed MAR algorithm, further evaluation of the algorithm using clinical data is required.

This study primarily investigated the impacts of MAR algorithms on HU value restoration on planning CT scans affected by metal artifacts. In addition to HU value restoration, the dosimetric impact of metal artifact reduction on the target (arrhythmogenic tissue) and OARs during the treatment planning is also crucial. Future work may therefore include an evaluation of the dosimetric impacts of the CCS-MAR algorithm.

Cone-Beam Computed Tomography (CBCT) is often used in radiation therapy, especially in the image-guided radiation therapy for patient positioning prior to the treatment [60]. This positioning is performed by matching the CBCT scans to the planning CT scan. Instead, or in addition to simultaneous acquisition of the planning CT scan and US images, simultaneous acquisition of CBCT scans and US images may be another way to implement US-based image guidance for cardiac radioablation. Therefore, the performance of the CCS-MAR algorithm should also be evaluated for transducer-induced metal artifact reduction on CBCT scans.

The application of deep learning for the metal artifact reduction on CT scans for the radiotherapy applications gained significant interest and has been actively studied in recent years [61–64]. These studies have shown promising results for the metal artifact reduction and claimed that the deep learning algorithms are powerful in handling the complex metal artifacts patterns, while preserving the anatomical features. Further, it was found that these algorithms often work on the image domain and thus create fewer number of secondary artifact and/or image blurring. Therefore, the potential application of deep learning for the reduction of the US transducer induced metal artifacts on CT scans for radiation therapy applications may be beneficial and worth exploring.

## Conclusion

The purpose of this work was to propose and evaluate an MAR algorithm which can be used to reduce metal artifacts resulting from the presence of a US transducer during CT scan acquisition. Visual comparison of CT scans, analysis of image quality metrics, and evaluation of the HU value restoration have shown that the proposed MAR algorithm, CCS-MAR, effectively reduces the negative impacts of US transducer-induced metal artifacts on planning CT scans of CT phantoms. These promising results justify further research into US transducer-induced MAR on CBCT scans as well as extensive (dosimetric) evaluations using clinical CT scans.
